# Studying C-reactive protein and D-dimer levels in blood may prevent severe complications: A study in Bangladeshi COVID-19 patients

**DOI:** 10.3389/fgene.2022.966595

**Published:** 2022-12-09

**Authors:** Gazi Nurun Nahar Sultana, Anshika Srivastava, Khalida Akhtaar, Prajjval Pratap Singh, Md. Anarul Islam, Rahul Kumar Mishra, Gyaneshwer Chaubey

**Affiliations:** ^1^ Centre for Advanced Research in Sciences (CARS), Genetic Engineering and Biotechnology Research Laboratory, University of Dhaka, Dhaka, Bangladesh; ^2^ Cytogenetics Laboratory, Department of Zoology, Banaras Hindu University, Varanasi, India

**Keywords:** CRP value, D-dimer, coronavirus, COVID-19, biomarker, Bangladesh

## Abstract

The ongoing COVID-19 pandemic has been a scientific, medical and social challenge. Since clinical course of this disease is largely unpredictable and can develop rapidly causing severe complications, it is important to identify laboratory biomarkers, which may help to classify patient’s severity during initial stage. Previous studies have suggested C—reactive protein (inflammatory) and D-dimer (biochemical) as an effective biomarker. The differential severity in patients across the world and our limited understanding in the progression of the disease calls for a multi-country analysis for biomarkers. Therefore, we have analyzed these biomarkers among 228 Bangladeshi COVID-19 patients. We observed significant association of COVID-19 severity with these two biomarkers. Thus, we suggest to use these biomarkers for Bangladeshi COVID-19 patients for better disease monitoring. Such validated preventive measures may decrease the case fatality ratio substantially.

## Introduction

COVID-19 patients are categorized into mild, moderate, severe, and critical based on disease severity. In order to stratify high risk patients there is an urgent need for reliable biomarkers related to coronavirus disease 2019 (COVID-19) progression. Novel biomarkers are also needed to understand viral pathogenetic mechanisms and to categorize patients into those who suffer rapid disease progression to severe complications like death. Association of severity with some hematological parameters like white blood cell (WBC), lymphopenia, and some biochemical parameters, such as Lactate dehydrogenase (LDH), creatine kinase (CK), IL-6 and troponin have already been reported to be associated with COVID-19 severity (Ponti et al., 2020).

C-reactive protein (CRP) is a pentameric protein, synthesized primarily in liver under the action of cytokine interleukin 6 (IL-6) (Ullah et al., 2020). Levels of CRP >40 mg/dl are reported to be associated with bacterial infections while more elevated levels are also seen in injuries, cardiovascular processes and other inflammatory states (Stringer et al., 2021). D-dimer is a fibrin degradation product (or FDP), a small protein fragment present in the blood after a blood clot is degraded by fibrinolysis (Adam et al., 2009). Higher D-dimer levels are indicative of the activation of coagulation and following fibrinolytic process (Adam et al., 2009) and, are reported in influenza like infections by respiratory viruses even before the outbreak of coronavirus pandemic (Van Wissen et al., 2011).

Although number of studies have already shown the association of COVID-19 severity with CRP and D-dimer levels in blood, there are limited studies done on Bangladeshi populations (Chowdhury et al., 2020; Khourssaji et al., 2020; Velavan and Meyer, 2020; Cabanillas et al., 2021; Synolaki et al., 2021). In a recent study done by (Ullah et al., 2020), both high D-Dimer (>501 ng/ml) and high CRP (>101 mg/dl) were associated with increased need for upgrade to the ICU and higher requirement for (intermittent mandatory ventilation) IMV on day-7 of hospitalization (Soraya and Ulhaq, 2020; Ullah et al., 2020). Therefore, for the first time in Bangladeshi individuals, plasma CRP and D dimer levels is demonstrated to assist for discerning patients with low to severe (live and dead) COVID-19 pneumonia belonging to different age groups. This suggest that these testing may be useful as an early indicator for severe illness and help physicians to stratify patients for intense care unit transfer (Chen et al., 2020). Our aim is to focus on CRP and D-dimer levels, which are potentially predictive of patients with severe complications and death in COVID-19 infection. In order to screen out biochemical indicators that are meaningful for the diagnosis of disease progression, we consulted the laboratory test results of all the dead and recovered patients from cabin/Ward and ICU.

## Methods

### Data collection

In this retrospective study, we include hematological data of all confirmed cases of COVID-19 from Anwar Khan Modern Medical College Hospital, Lab Aid Medical College Hospital, and in some cases communication with patient’s family from June 2020 to November 2020. The patients in this dataset were not vaccinated. Ethical permission was obtained from the Biological Science Faculty, Dhaka University, Bangladesh. The medical treatment consent was obtained by the physicians of the two Hospitals. We have called few patients (or their family) and explained this study to them to obtain their consent. COVID-19 test were done using reverse transcriptase polymerase chain reaction (RT-PCR) technique. We have accessed to patients’ data at the end of November 2020, only with serial number (fully anonymous). We include those patients with criteria having CRP and D-dimer results and admitted to ICU and non-ICU. We considered 144 cases from ICU, 84 cases from non-ICU. We have communicated with patients’ relatives to get missing information. The patients’ lab test data also include age, gender, patients’ status and hospital status.

All medical laboratory data including the concentrations of D-dimer, and high-sensitivity C-reactive protein (CRP), were generated by the Biochemistry laboratory of two Hospitals recorded electronically in their database. The samples for laboratory tests were collected on admission and during the hospital stay or visit. Peripheral venous blood was collected and D-dimer was measured with a latex particle-enhanced immune turbidimetric assay Sysmex 1,000 (Siemens Healthcare, NY, United States). It is worth mentioning that CRP tests were detected using Dimensional RxL Max integrated chemistry system (Siemens Healthcare, NY, United States) in both hospitals. Supplementary Table S3 gives descriptive characteristics for our dataset.

### Statistical analysis

CRP and D-Dimer levels were collected from the patients admitted in Cabin/Ward and ICU. for Spearman correlation coefficient or Kendall’s tau-b correlation coefficient tests were applied to test the correlation among patient’s age, hospital status, D-dimer and CRP value and patient’s status (Survived or Died) using SPSS v 26.0.0.0. We recoded patient recovered and dead status as 0 and 1 respectively. Similarly, male and female sexes were coded as 1 and 0 respectively. Hospital Cabin/Ward patients and ICU patients were coded into 0 and 1. We ran Logistic Regression Test for the combined dataset keeping Severity as dependent variable and CRP and D-dimer levels as independent while adjusting with age and gender. We categorized CRP levels >40 mg/L as elevated and <40 mg/L as Normal. A *p* value below 0.05 was considered statistically significant.

## Results and discussion

Inflammatory responses play a crucial role in progression of COVID-19 (García, 2020; Merad and Martin, 2020; Tufan et al., 2020). Rapid viral replication of the SARS-CoV-2 involves recruitment of macrophages and monocytes, release of cytokines and chemokines, thus triggering inflammatory responses (Tay et al., 2020). CRP (C-Reactive protein) has been reported in dengue patients (Vuong et al., 2020). Dengue virus and SARS-CoV-2 are RNA virus, share similarity in the course of infection (Vuong et al., 2020). CRP is rapidly synthesized in response to a variety of eukaryotic and prokaryotic pathogens, by hepatocytes when stimulated by inflammation, facilitating complement activation through classical pathway (Mold et al., 1999), indicating immune activation, lymphocyte infiltration, immune molecules consumption and inflammation outbreak. It is also inferred that increased CRP levels could be early indicators of nosocomial infections in COVID-19 patients who were slow to recover (Chen et al., 2020). Higher levels of CRP, a potential inflammatory biomarker has been reported to be significantly associated with disease severity in COVID-19 infections (Chen et al., 2020; Gao et al., 2020; Qin et al., 2020). CRP levels have also been reported to be increased in elderly or old age individuals (Wyczalkowska-Tomasik et al., 2016; De Almeida Roediger et al., 2019). Elevated baseline CRP predicted IMV or death (Temesgen et al., 2022a). Lenzilumab is a brand-new anti-human GM-CSF monoclonal antibody that binds to GM-CSF directly to stop any downstream signaling (Temesgen et al., 2022b). Several findings have reported that patients with elevated CRP (>150 mg/l) may benefit from lenzilumab (Temesgen et al., 2022a; Kilcoyne et al., 2022).

**FIGURE 1:**
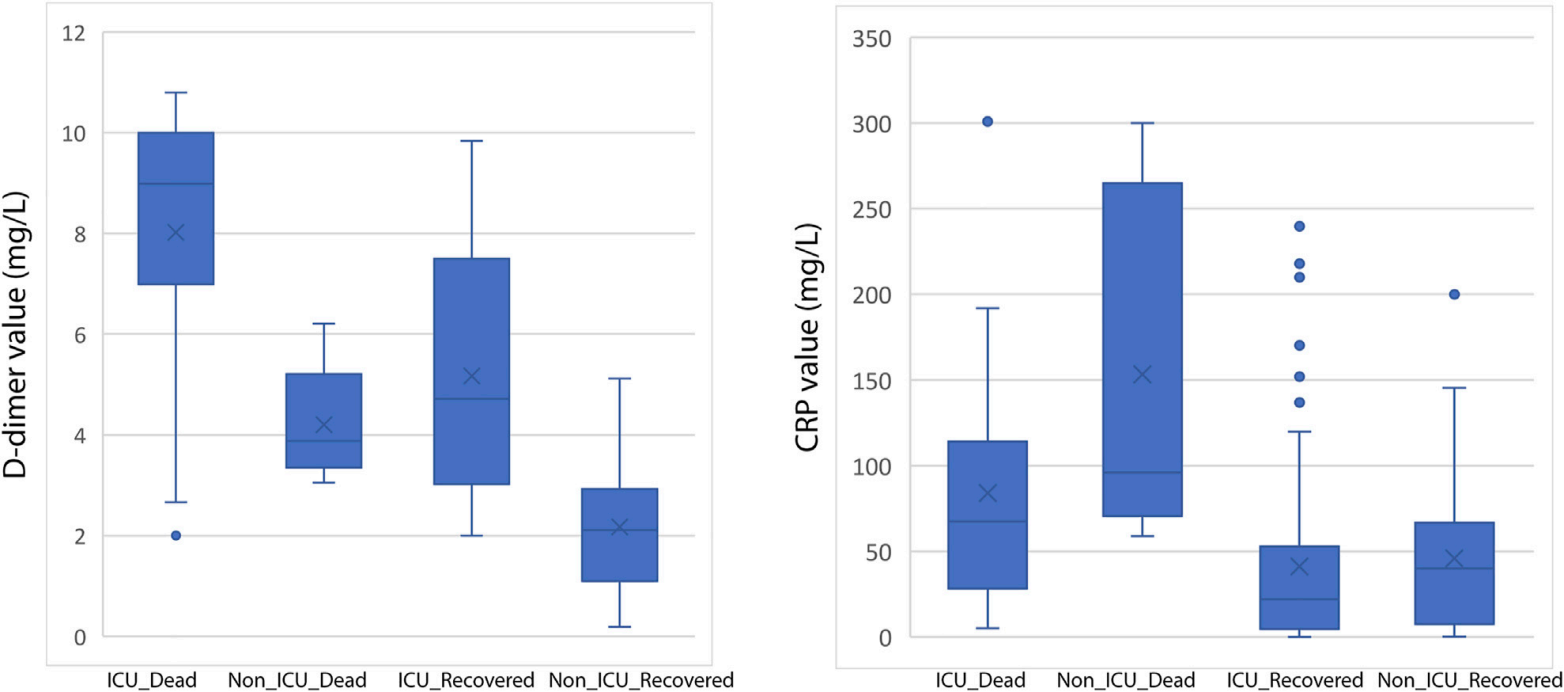
Box-and-whisker plot showing distribution of D-dimer (left) and CRP (right) values among various categories of COVID-19 patients.

A total of 228 patients with a confirmed diagnosis of COVID-19 were included in our study. (Supplementary Tables S1, S2). Based on disease severity, patients were divided into two comparison groups (Cabin/Ward and ICU patients). We found significantly strong correlation between D-dimer and CRP levels with age and Mortality in the combined dataset of ICU and non-ICU COVID-19 patients (Figure 1 and Table 1). Increased age and D-dimer values were found to be significantly associated with the patients admitted to ICU. Significant correlation of CRP levels with Age (Correlation coefficient = 0.250, *p*-value < 0.05) was found in non-ICU patients (Supplementary Table S4). In patients admitted to ICU, we found strong correlation of D-dimer with age (Correlation Coefficient = 0.304, *p*-value < 0.01). Both Higher D-dimer (odds ratio = 1.726, 95% confidence interval: 1.420–2.089, *p* < 0.001) and CRP-values (odds ratio = 5.436 95% confidence interval: 1.995128–14.811197, *p* < 0.001) were associated with increased mortality in patients (Table 2). We also showed age was associated with elevated D-dimer and that higher D-dimer value was associated with mortality (>8.99 mg/L). (Supplementary Table S6). We found significant *p* values for Welch T test for comparing Patient’s status i. e mortality with CRP, D-dimer value and age (Supplementary Table S10).

**TABLE 1:** Correlations between Age, Gender, D-dimer value, CRP value, with Mortality for the combined dataset of ICU and non-ICU COVID-19 patients.

Variables	Spearman coefficient	Kendall Tau’s coefficient
Age	**0.213 (*p* < 0.01)**	0.**176(*p* < 0.01)**
Gender	0.033 (*p* >0.05)	0.033 (*p* >0.05)
CRP (mg/ml)	**0.310 (*p* < 0.01)**	**0.254 (*p* < 0.01)**
D dimer (mg/ml)	**0.442(*p* < 0.01)**	0.**363 (*p* < 0.01)**

The bold values indicate the significance of the correlation tests at *p* < 0.01.

**TABLE 2:** Logistic regression analysis predictive of mortality in COVID-19 patients.

Variable	Odds ratio (95% CI)	*p* value
CRP (mg/l)	5.436 (1.995128–14.811197)	**< 0.001**
D-dimer (μg/ml)	1.726 (1.427523-2.087940)	**<0.001**
Gender	1.650 (0.569- 4.786288)	0.357
Age	1.020 (0.976365-1.065480)	0.375

The bold values indicate the significance of the correlation tests at *p* < 0.001.

It is interesting to note that D-dimer is commonly high in patients with COVID-19. In patients who were admitted for COVID-19 treatment, D-dimer levels are a reliable predictive test to look for the occurrence of a major blood clot. While higher levels of C reactive protein (CRP) may be a predictive in marker determining systemic inflammation and can predict which patients with mild COVID-19 will progress to a severe case.

Processes that involve production and breakdown of fibrin cause an elevation in D-dimer levels (Simes et al., 2018; Ullah et al., 2020). Increased D dimer levels are reported to develop acute respiratory distress in COVID-19, with the more chances of micro pulmonary embolism especially in severe forms of COVID-19 (Vidali et al., 2020). D-dimer levels have been reported to vary among patients with confirmed venous thromboembolism (VTE) depending on clot burden, timing of measurement, and initiation of treatment (Linkins and Takach Lapner, 2017). D-dimer has also been shown to increase with age, which can cause more false positive tests in older patients (Kabrhel et al., 2010). Also, several potential risk factors during hospitalization like, disseminated intra vascular coagulation, infection, dehydration, prolonged immobilization, mechanical ventilation, and central venous catheter use may further increase D-dimer concentrations (Terpos et al., 2020; Wang et al., 2020).

Several studies have revealed a startling correlation between ethnicity and D-dimer levels, with African Americans having the highest levels and Asians having the lowest (Pieper et al., 2000; Chaudhary et al., 2020). Additionally, CRP levels have been found to be greater in ethnic minority individuals of South Asian Surinamese, African Surinamese, Turkish, and Moroccan descent than Dutch participants, but similar among Ghanaians (Muilwijk et al., 2019). These findings suggest that there may be ethnic differences in low-grade inflammation. In conclusion, we confirm the association of two main, inflammatory and biochemical covariates with COVID-19 severity for the first time in Bangladeshi patients. The study can help in detail understanding of the complications caused and predict the progression of the disease with much more confidence.

## Data Availability

The original contributions presented in the study are included in the article/Supplementary Materials, further inquiries can be directed to the corresponding author.
