# Supplementary material for: Studying C-reactive protein and D-dimer levels in blood may prevent severe complications: A study in Bangladeshi COVID-19 patients
Source: Front Genet. 2022 Dec 9;13:966595. doi: 10.3389/fgene.2022.966595 (PMC9780378; doi:10.3389/fgene.2022.966595)
Supplement: Supplementary file 1 [file DataSheet1.PDF]

Supplementary data

Supplementary Table 1. Values of D-dimer and CRP in ICU patients admitted to Hospital.

Patient-ID	Gender	Age	Hospital status	D-dimer Value (mg/L)	CRP value (mg/L	Patient's status
Cov-01	M	70	ICU/HDU	5.86	120	Survived
Cov-02	M	60	ICU/HDU	2.99	12	Survived
Cov-03	F	54	ICU/HDU	8.5	40	Died
Cov-04	F	58	ICU/HDU	4.33	50	Survived
Cov-05	M	62	ICU/HDU	3.42	3	Survived
Cov-06	M	80	ICU/HDU	>7.50	3.33	Survived
Cov-07	F	72	ICU/HDU	>7.27	192	Died
Cov-08	M	68	ICU/HDU	2.28	3	Survived
Cov-09	M	61	ICU/HDU	>7.50	5	Survived
Cov-10	F	50	ICU/HDU	7.81	218	Survived
Cov-11	M	61	ICU/HDU	2.54	22	Survived
Cov-12	M	64	ICU/HDU	4.84	95	Survived
Cov-13	M	71	ICU/HDU	8.05	6	Survived
Cov-14	F	49	ICU/HDU	2.83	61	Died
Cov-15	M	62	ICU/HDU	7.15	3	Survived
Cov-16	M	67	ICU/HDU	7.15	53	Survived
Cov-17	M	61	ICU/HDU	6.38	99	Survived
Cov-18	M	49	ICU/HDU	7.62	60	Survived
Cov-19	F	57	ICU/HDU	7.5	95	Survived
Cov-20	M	48	ICU/HDU	3.48	16	Survived
Cov-21	M	64	ICU/HDU	7.25	34	Survived
Cov-22	M	70	ICU/HDU	3.77	8	Survived
Cov-23	M	42	ICU/HDU	2.85	42	Survived
Cov-24	M	59	ICU/HDU	2.77	62	Survived
Cov-25	M	56	ICU/HDU	>7.50	8	Survived
Cov-26	F	60	ICU/HDU	5	42	Survived
Cov-27	M	53	ICU/HDU	3.03	21	Survived
Cov-28	M	46	ICU/HDU	2.08	54	Survived
Cov-29	M	70	ICU/HDU	2.87	22	Survived
Cov-30	M	69	ICU/HDU	7.5	5.1	Survived
Cov-31	M	53	ICU/HDU	5.93	6.7	Survived
Cov-32	M	60	ICU/HDU	7.21	72	Died
Cov-33	M	62	ICU/HDU	>7.50	33.9	Survived
Cov-34	M	60	ICU/HDU	3.39	170.2	Survived

Cov-35	M	70	ICU/HDU	7.25	301	Died
Cov-36	M	48	ICU/HDU	3.72	4.7	Survived
Cov-37	F	52	ICU/HDU	8.5	119	Survived
Cov-38	M	?	ICU/HDU	3.42	62	Survived
Cov-39	F	48	ICU/HDU	2.4	210	Survived
Cov-40	F	40	ICU/HDU	7.5	11	Survived
Cov-41	M	60	ICU/HDU	5.09	1	Survived
Cov-42	M	?	ICU/HDU	6.07	37	Survived
Cov-43	M	60	ICU/HDU	>7.50	19	Died
Cov-44	M	71	ICU/HDU	7.5	8.2	Survived
Cov-45	M	70	ICU/HDU	7.11	8	Survived
Cov-46	M	60	ICU/HDU	6.26	5	Survived
Cov-47	F	62	ICU/HDU	3	5	Survived
Cov-48	M	70	ICU/HDU	2.57	17.6	Survived
Cov-49	M	64	ICU/HDU	6.53	7.2	Survived
Cov-50	M	59	ICU/HDU	10.8	113.4	Died
Cov-51	M	60	ICU/HDU	7.5	5.4	Survived
Cov-52	M	58	ICU/HDU	2.2	137	Survived
Cov-53	M	70	ICU/HDU	7.9	2	Survived
Cov-54	M	68	ICU/HDU	7.7	4.7	Survived
Cov-55	M	60	ICU/HDU	8.5	138	died
Cov-56	M	66	ICU/HDU	4.02	4.6	Survived
Cov-57	M	60	ICU/HDU	9.84	-	Survived
Cov-58	M	40	ICU/HDU	2.38	2.2	Survived
Cov-59	M	51	ICU/HDU	5.16	50.4	Survived
Cov-60	M	60	ICU/HDU	4.61	100.4	Died
Cov-61	M	66	ICU/HDU	8.5	214	Survived
Cov-62	M	70	ICU/HDU	8.5	138.7	Survived
Cov-63	M	46	ICU/HDU	2.8	23.2	Survived
Cov-64	M	49	ICU/HDU	7.5	51.1	Survived
Cov-65	F	40	ICU/HDU	3.42	26.9	Survived
Cov-66	M	85	ICU/HDU	3.7	-	Survived
Cov-67	F	82	ICU/HDU	7.05	239.7	Survived
Cov-68	F	72	ICU/HDU	8.5	2	Survived
Cov-69	F	82	ICU/HDU	8.9	3.8	Survived
Cov-70	F	80	ICU/HDU	9	1	Survived
Cov-71	F	76	ICU/HDU	7.51	37.8	Died
Cov-72	F	48	ICU/HDU	4.09	2.5	Survived
Cov-73	M	71	ICU/HDU	4.3	3	Survived
Cov-74	M	77	ICU/HDU	4.38	21.3	Survived
Cov-75	M	58	ICU/HDU	4.6	0	Survived

Cov-76	M	47	ICU/HDU	4.9	14.5	Survived
Cov-77	M	69	ICU/HDU	4.96	152	Survived
Cov-78	M	66	ICU/HDU	5.32	85	Died
Cov-79	F	62	ICU/HDU	6.3	71	Survived
Cov-80	F	49	ICU/HDU	6.5	0.99	Survived
Cov-81	M	56	ICU/HDU	6.88	3.84	Survived
Cov-82	F	62	ICU/HDU	7.88	102	Survived
Cov-83	F	56	ICU/HDU	8.26	62	Survived
Cov-84	M	78	ICU/HDU	8.79	3.7	Survived
Cov-85	M	74	ICU/HDU	8.81	5.2	Died
Cov-86	M	69	ICU/HDU	9.17	111	Died
Cov-87	M	62	ICU/HDU	9.69	103	Died
Cov-88	M	82	ICU/HDU	9.94	134.2	Died
Cov-89	M	63	ICU/HDU	10	56.3	Died
Cov-90	M	49	ICU/HDU	10	47.9	Died
Cov-91	M	58	ICU/HDU	10	12.8	Died
Cov-92	M	69	ICU/HDU	10	24.3	Died
Cov-93	M	70	ICU/HDU	10	22.9	Died
Cov-94	M	78	ICU/HDU	10	172	Died
Cov-95	M	61	ICU/HDU	10	155	Died
Cov-96	M	59	ICU/HDU	10	103	Died
Cov-97	F	72	ICU/HDU	10	28	Died
Cov-98	F	66	ICU/HDU	10	63	Died
Cov-99	M	78	ICU/HDU	10.62	51	Died
Cov-100	M	71	ICU/HDU	6.33	28.4	Died
Cov-101	F	49	ICU/HDU	2	17.6	Died
Cov-102	F	44	ICU/HDU	2	22.3	Survived
Cov-103	F	54	ICU/HDU	2	38.5	Survived
Cov-104	M	67	ICU/HDU	2	55.7	Survived
Cov-105	M	66	ICU/HDU	2.87	52.1	Survived
Cov-106	M	73	ICU/HDU	4	34.7	Survived
Cov-107	F	70	ICU/HDU	4	31.2	Survived
Cov-108	M	68	ICU/HDU	4.07	49	Survived
Cov-109	M	60	ICU/HDU	4.1	109	Died
Cov-110	F	49	ICU/HDU	2.66	117	Died
Cov-111	F	?	ICU/HDU	2.8	26	Survived
Cov-112	F	38	ICU/HDU	2.85	31	Survived
Cov-113	F	56	ICU/HDU	2.9	1.9	Survived
Cov-114	F	62	ICU/HDU	2.9	3.5	Survived
Cov-115	M	67	ICU/HDU	2.92	22.5	Survived
Cov-116	M	62	ICU/HDU	3.02	71	Survived

Cov-117	M	81	ICU/HDU	3.45	52	Survived
Cov-118	M	73	ICU/HDU	3.5	5.6	Survived
Cov-119	M	66	ICU/HDU	3.8	3.2	Survived
Cov-120	M	82	ICU/HDU	5.16	2.2	Survived
Cov-121	M	58	ICU/HDU	3.22	34	Survived
Cov-122	M	72	ICU/HDU	4.26	16	Survived

Supplementary Table 2. Values of D-dimer and CRP in non-ICU patients admitted to Hospital.

Serial Number	Gender	Age	Cabin/Ward	D-dimer valu (mg/L	CRP value (mg/L)	Patient's status
Cov-01	M	80	Cabin/Ward	0.92	28	survived
Cov-02	M	58	Cabin/Ward	1.44	46	survived
Cov-03	M	39	Cabin/Ward	1.42	70	survived
Cov-04	M	60	Cabin/Ward	1.49	66	survived
Cov-05	M	24	Cabin/Ward	2.11	44	survived
Cov-06	F	47	Cabin/Ward	2.98	12	survived
Cov-07	M	53	Cabin/Ward	3.05	96	Died
Cov-08	F	29	Cabin/Ward	1.49	96	survived
Cov-09	M	71	Cabin/Ward	4.21	82	Died
Cov-10	M	26	Cabin/Ward	2.36	102	survived
Cov-11	M	65	Cabin/Ward	4	32	survived
Cov-12	M	76	Cabin/Ward	0.98	24	survived
Cov-13	M	96	Cabin/Ward	0.92	94	survived
Cov-14	M	38	Cabin/Ward	0.99	40	survived
Cov-15	F	41	Cabin/Ward	1.32	40	survived
Cov-16	F	50	Cabin/Ward	2.14	46	survived
Cov-17	M	48	Cabin/Ward	2.89	93.6	survived
Cov-18	M	60	Cabin/Ward	0.91	50	survived
Cov-19	M	48	Cabin/Ward	0.19	49	survived
Cov-20	M	60	Cabin/Ward	0.78	56	survived
Cov-21	M	32	Cabin/Ward	2.37	62.8	survived
Cov-22	M	43	Cabin/Ward	2.14	21.1	survived

Cov-23	M	36	Cabin/Ward	1.98	5	survived
Cov-24	F	55	Cabin/Ward	3.66	52	survived
Cov-25	M	71	Cabin/Ward	6.21	59	Died
Cov-26	M	12	Cabin/Ward	1.96	24.1	survived
Cov-27	M	43	Cabin/Ward	2.85	55.8	survived
Cov-28	M	63	Cabin/Ward	2.54	5	survived
Cov-29	M	55	Cabin/Ward	2.96	74.4	survived
Cov-30	M	71	Cabin/Ward	2.44	205	survived
Cov-31	M	22	Cabin/Ward	2.35	1	survived
Cov-32	M	41	Cabin/Ward	2	-	survived
Cov-33	F	63	Cabin/Ward	2.88	145.4	survived
Cov-34	F	40	Cabin/Ward	3.45	6.1	survived
Cov-35	F	63	Cabin/Ward	4	200	survived
Cov-36	F	42	Cabin/Ward	2.55	117	survived
Cov-37	M	66	Cabin/Ward	3.96	86.4	survived
Cov-38	M	54	Cabin/Ward	3.12	79.5	survived
Cov-39	M	77	Cabin/Ward	2.18	80.7	survived
Cov-40	M	61	Cabin/Ward	1.66	23.7	survived
Cov-41	M	59	Cabin/Ward	2.6	0.59	survived
Cov-42	M	64	Cabin/Ward	2.85	80	survived
Cov-43	M	?	Cabin/Ward	3.77	71	survived
Cov-44	M	46	Cabin/Ward	3.21	23.8	survived
Cov-45	M	36	Cabin/Ward	3.82	1.23	survived
Cov-46	M	52	Cabin/Ward	2.56	40	survived
Cov-47	M	73	Cabin/Ward	2.99	55.8	survived
Cov-48	F	63	Cabin/Ward	1.87	35	survived
Cov-49	M	29	Cabin/Ward	0.9	63	survived
Cov-50	F	38	Cabin/Ward	1.04	0.28	survived
Cov-51	F	81	Cabin/Ward	0.99	11.6	survived
Cov-52	M	24	Cabin/Ward	0.88	42	survived
Cov-53	M	40	Cabin/Ward	1.54	4	survived
Cov-54	M	63	Cabin/Ward	2.36	2.3	survived
Cov-55	M	35	Cabin/Ward	4.52	0.98	survived
Cov-56	F	?	Cabin/Ward	2.55	3.56	survived

Cov-57	F	44	Cabin/Ward	2.16	2.7	survived
Cov-58	F	62	Cabin/Ward	3.26	45	survived
Cov-59	F	36	Cabin/Ward	4.22	21	survived
Cov-60	F	63	Cabin/Ward	3.98	9	survived
Cov-61	M	38	Cabin/Ward	2.99	97	survived
Cov-62	M	79	Cabin/Ward	1.56	37	survived
Cov-63	M	48	Cabin/Ward	1.33	107	survived
Cov-64	M	60	Cabin/Ward	0.89	36	survived
Cov-65	M	24	Cabin/Ward	1.71	14	survived
Cov-66	M	18	Cabin/Ward	0.85	50	survived
Cov-67	M	62	Cabin/Ward	4.15	26	survived
Cov-68	M	34	Cabin/Ward	1.63	37	survived
Cov-69	M	49	Cabin/Ward	2.66	104	survived
Cov-70	F	42	Cabin/Ward	4.12	84	survived
Cov-71	M	56	Cabin/Ward	1.11	67	survived
Cov-72	M	82	Cabin/Ward	3.05	120	survived
Cov-73	M	28	Cabin/Ward	0.97	6	survived
Cov-74	M	52	Cabin/Ward	3.88	300	Died
Cov-75	M	41	Cabin/Ward	2.47	4	survived
Cov-76	M	49	Cabin/Ward	2.1	40.7	survived
Cov-77	F	44	Cabin/Ward	5.12	43.5	survived
Cov-78	F	61	Cabin/Ward	3.21	90	survived
Cov-79	M	58	Cabin/Ward	3.28	1.2	survived
Cov-80	M	55	Cabin/Ward	2.22	0.87	survived
Cov-81	F	43	Cabin/Ward	3.99	1.32	survived
Cov-82	F	65	Cabin/Ward	0.86	27.3	survived
Cov-83	F	62	Cabin/Ward	0.78	7.2	survived
Cov-84	F	19	Cabin/Ward	2.33	16.6	survived
Cov-85	M	45	Cabin/Ward	1.03	32	survived
Cov-86	M	82	Cabin/Ward	1.08	5	survived
Cov-87	M	67	Cabin/Ward	3.22	55	survived
Cov-88	M	26	Cabin/Ward	1.03	32	survived
Cov-89	F	42	Cabin/Ward	1	48	survived
Cov-90	M	57	Cabin/Ward	1.83	60	survived

Cov-91	M	61	Cabin/Ward	0.94	110.7	survived
Cov-92	M	66	Cabin/Ward	0.83	25.1	survived
Cov-93	M	48	Cabin/Ward	0.7	32	survived
Cov-94	M	27	Cabin/Ward	1.92	42	survived
Cov-95	M	36	Cabin/Ward	0.94	51	survived
Cov-96	M	62	Cabin/Ward	1.35	0.98	survived
Cov-97	F	44	Cabin/Ward	1.93	57	survived
Cov-98	M	27	Cabin/Ward	1.88	1.22	survived
Cov-99	M	52	Cabin/Ward	2.36	130	survived
Cov-100	M	80	Cabin/Ward	0.75	122	survived
Cov-101	M	51	Cabin/Ward	1.99	2.69	survived
Cov-102	M	44	Cabin/Ward	2.54	9	survived
Cov-103	F	29	Cabin/Ward	2.37	0.21	survived
Cov-104	M	34	Cabin/Ward	2.11	5	survived
Cov-105	M	56	Cabin/Ward	2.08	0.93	survived
Cov-106	M	73	Cabin/Ward	3.66	230	Died

Supplementary Table 3. Descriptive characteristics for ICU and Non-ICU patients with comparison by in-hospital mortality

	**All Patients(228)**	**Dead(35)**	**Alive(193)**	Male	Female	**Age(<60)**	**Age(>60)**	**Samples with Missing info**
ICU	122(53.5%)	30(24.6%)	92(75.4%)	89(73%)	33(27%)	40(32.8%)	67(55%)	5(4%)
Non-ICU	106(46.5%)	5(4.71%)	101(95.3%)	79(74.5%)	27(25.5%)	67(63.2%)	33(31.1%)	3(3%)

Supplementary Table 4. Correlations between Age, D-dimer value and CRP value for non-ICU COVID-19 patients

		Age	CRP value (mg/L)	D-dimer valu (mg/L)
Age	Pearson Correlation	1	.250*	0.071
	Sig. (2-tailed)		**0.011**	0.476
	N	104	103	104
CRP value (mg/L)	Pearson Correlation	.250*	1	0.19
	Sig. (2-tailed)	**0.011**		0.052
	N	103	105	105
D-dimer valu (mg/L	Pearson Correlation	0.071	0.19	1
	Sig. (2-tailed)	0.476	0.052	
	N	104	105	106

Supplementary Table 5. Correlations between Age, D-dimer value and CRP value, for ICU COVID-19 patients

		**Age**	**CRP value (mg/L**	**D-dimer Value (mg/L)**
**Age**	Pearson Correlation	1	0.055	.304**
	Sig. (2-tailed)		0.558	**0.001**
	N	119	117	119
**CRP value (mg/L)**	Pearson Correlation	0.055	1	.193*
	Sig. (2-tailed)	0.558		**0.034**
	N	117	120	120
**D-dimer Value (mg/L)**	Pearson Correlation	.304**	.193*	1
	Sig. (2-tailed)	**0.001**	**0.034**	
	N	119	120	122

Supplementary Table 6. Spearman's rho and Kendall's tau b correlation tests between Age, D-dimer value and CRP value for the combined dataset of ICU and non-ICU COVID-19 patients.

	**Age**	**D-dimer value (mg/L**	**CRP value (mg/L)**

Kendall's tau_b	**Age**	Correlation Coefficient	1.000	.281**	.060
		Sig. (2-tailed)	.	**<0.01**	.188
		N	223	223	220
	**D-dimer valu (mg/L**	Correlation Coefficient	.281**	1.000	.039
		Sig. (2-tailed)	**<0.01**	.	.381
		N	223	228	225
	**CRP value (mg/L)**	Correlation Coefficient	.060	.039	1.000
		Sig. (2-tailed)	.188	.381	.
		N	220	225	225
Spearman's rho	**Age**	Correlation Coefficient	1.000	.394**	.086
		Sig. (2-tailed)	.	**<0.01**	.205
		N	223	223	220
	**D-dimer valu (mg/L**	Correlation Coefficient	.394**	1.000	.065
		Sig. (2-tailed)	**<0.01**	.	.334
		N	223	228	225
	**CRP value (mg/L)**	Correlation Coefficient	.086	.065	1.000
		Sig. (2-tailed)	.205	.334	.
		N	220	225	225

Supplementary Table 7. Results of Pearson Chi Square Test between Age, Sex, Patient's Status (Severity) and Ward (ICU/Non- ICU) for the combined dataset of ICU and non-ICU COVID-19 patients.

**Variables Compared**	**Fisher's Exact Test P value**

**Sex * Ward (ICU/Non-ICU)**	0.757
**Sex * Patients' status(Severity)**	0.525

Supplementary Table 8. Welch T test for comparing Gender with D-Dimer and CRP value in ICU and Non-ICU patients.

Variables	P value
**D-dimer**	0.982
**CRP**	0.569

Supplementary Table 9. Welch T test for comparing Ward (ICU/Non-ICU) with D-Dimer, CRP value and Age in ICU and Non-ICU patients.

Variables	P value
D-Dimer	0.805
CRP Value	<0.001
Age	<0.001

Supplementary Table 10. Welch T test for comparing Patient's status (Live or Dead) with D-Dimer, CRP value and Age in ICU and Non-ICU patients.

Variables	P value
D-Dimer	<0.01
CRP Value	<0.001
Age	<0.001

Supplementary Table 11. Median values for D dimer and CRP levels in all groups of ICU and Non-ICU COVID-19 Patients.

Groups	D dimer value	CRP value
ICU_dead	8.99	67.5
ICU_Recovered	4.72	22
NON_ICU_Recovered	2.11	40
NonICU_Dead	3.88	96
